# Hybrid Bismuth Halide with Rich Polymorphism and Second Harmonic Generation Response

**DOI:** 10.1021/acsmaterialslett.5c00784

**Published:** 2025-07-07

**Authors:** Aleksandra D. Valueva, Sergei A. Novikov, Eric Gabilondo, Hunter B. Tisdale, Alevtina A. Maksimova, Mikhail Parker, Vladimir Reukov, Vladislav V. Klepov

**Affiliations:** † Department of Chemistry, 1355 University of Georgia, Athens, Georgia 30602, United States; ‡ Department of Chemistry, 14743 University of Houston, Houston, Texas 77204, United States; § Department of Chemistry, 2629 University of South Carolina, Columbia, South Carolina 29208, United States; ∥ Textiles, Merchandising and Interiors, University of Georgia, Athens, Georgia 30605, United States

## Abstract

Hybrid structures have emerged as a promising class of optical materials, due to their ability to couple the robustness of inorganic and the tunability of organic compounds. However, their application in nonlinear optics (NLO) remains limited, largely due to underexplored factors that drive the formation of noncentrosymmetric structures and NLO property characterization. In this work, we explore the formation, structural and temperature polymorphism, and optical properties of the (Et_3_NH)_3_Bi_2_Br_9_ composition, which crystallizes as either noncentrosymmetric or centrosymmetric polymorph. Structural analysis showed that the alignment of [Bi_2_Br_9_]^3–^ units dictates the symmetry of phases, as well as the nonlinear optical properties, with the triclinic polymorph exhibiting a second harmonic generation (SHG) response both in visible and IR regions (1.29 × KH_2_PO_4_ and 0.08 × AgGaS_2_). Thermal analysis reveals polymorphic phase transitions and low melting points, making them melt-processable and ionic liquid candidates.

## Graphical abstract

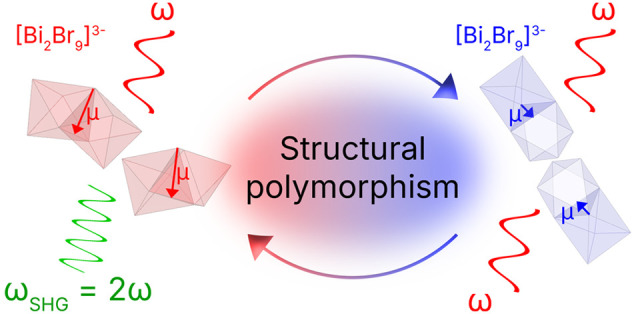

Hybrid materials garner increasing research attention due to their diverse properties and structures.^1^ The combination of highly tunable organic cations with robust inorganic fragments enables property control and favors these materials for potential applications as light-emitting diodes (LEDs),^2–7^ solar cells,^8–14^ photo- and radiation detectors,^15–19^ and lasers.^20–22^ For example, the size of organic cations plays an important role in the photoluminescent properties of these materials, as illustrated by manganese, indium, and tellurium hybrid materials.^23–25^ On the other hand, the structure of metal halide building units can be tuned to alter the electronic properties. While isolated octahedral units containing *n*s^2^ cations favor photoluminescent properties, offering high quantum yields and lifetimes,^26–30^ metal halide building units with higher dimensionalities promote increased charge carrier mobilities.^31,32^ Since both metal halide and organic building units play an important role in defining the characteristics of the resulting phase, their careful selection and matching are crucial in achieving the desirable properties of such materials. Recently, this ability to merge the advantages of both organic and inorganic components has made hybrid materials promising candidates in the field of nonlinear optics (NLO), where careful structural design can promote desirable NLO properties.

Second harmonic generation (SHG) materials that enable the generation of light with doubled frequency have found many practical applications in various fields, ranging from medicine to home defense.^33–35^ An essential requirement for SHG is noncentrosymmetric crystal structure, as the SHG coefficient is zero otherwise. SHG materials are typically categorized by their intended spectral application: infrared (IR), visible range, and ultraviolet (UV). Commercially available inorganic NLO materials exist for both IR applications (e.g., AgGaS_2_ (AGS), AgGaSe_2_ (AGSe),^36–38^ and ZnGeP_2_ ^39,40^ ) and UV–visible applications (e.g., KH_2_PO_4_ (KDP),^41,42^ KTiOPO_4_ (KTP), and their families,^43^ borates such as LiB_3_O_5_ (LBO) and CsB_3_O_5_ (CBO),^44,45^ metal iodates,^46,47^ carbonates, nitrates, phosphates^48–50^ ), but each group has notable limitations.^51–54^ Inorganic NLO materials offer high thermal stability, high laser-induced damage thresholds (LIDT), and good mechanical properties,^55–60^ whereas organic molecules typically exhibit much stronger SHG responses but suffer from poor thermal and mechanical stability.^61,62^ Hybrid halide materials, combining advantageous properties of both organic and inorganic materials, are therefore highly promising candidates for both IR and UV–visible SHG applications. Careful structural design and the strategic selection of organic cations and metal halide units can promote noncentrosymmetry and optimize performance across different spectral ranges. Despite this potential, reports on hybrid metal halide SHG materials, especially those operating effectively across multiple spectral regions, remain limited, emphasizing the importance of further research.

In this report, we studied the formation of noncentrosymmetric hybrid materials in the Et_3_NH^+^–Bi^3+^–Br^–^ system and their application for SHG. We crystallized two phases with the same chemical composition (Et_3_NH)_3_Bi_2_Br_9_, which exhibit non- and centrosymmetric structures. The structural difference in those phases stems from the different mutual arrangements of noncentrosymmetric [Bi_2_Br_9_]^3–^ structural units in the unit cells. Interestingly, (Et_3_NH)_3_Bi_2_Br_9_ exhibits both temperature and metastable polymorphism, showing a complex energetic landscape of structural stabilities in this composition. SHG measurements revealed that the noncentrosymmetric polymorph exhibits a modest SHG response of 0.08 × AGS in the IR region; importantly, this polymorph is phase-matchable, preserving its potential practical relevance. Additionally, we explored the visible region SHG properties of this polymorph, which demonstrated a notable SHG response of 1.29 × KDP, although it was found to be non-phase-matchable. Thermal analysis revealed very low melting points of (Et_3_NH)_3_Bi_2_Br_9_ and its Sb analogue, which renders them melt-processable materials. Overall, this work significantly advances the knowledge of hybrid SHG materials and highlights the importance of their further development for potential nonlinear optical applications.

We synthesized two phases with the same composition of (Et_3_NH)_3_Bi_2_Br_9_ via evaporation of aqueous solutions. Starting materials bismuth­(III) oxide and triethylamine were dissolved in HBr (56 wt %), which also served as a Br^–^ source. After complete dissolution, the vials were placed into an oven at 95 °C for 1 h to concentrate the starting solution. The final crystallization step was done by either slow evaporation at room temperature or by rapid evaporation in an oven at 95 °C. Slow evaporation results in a phase pure sample of triclinic (Et_3_NH)_3_Bi_2_Br_9_ phase (Figure S1a), whereas fast evaporation results in a mixture of triclinic and monoclinic polymorphs of (Et_3_NH)_3_Bi_2_Br_9_ (Figure S1b). Due to the apparent stability under slow evaporation conditions, the triclinic phase can be obtained in the form of large crystals of high quality. The size of the crystals depends on the crystallization conditions, and undisturbed solutions with limited crystallization centers tend to result in bigger crystals up to 4–5 mm. Our attempts to obtain large, high-quality single crystals of the monoclinic phase did not succeed due to the crucial role of fast evaporation in their formation, which simultaneously promotes crystal growth on multiple crystallization centers, resulting in smaller single crystals. For the synthesis of triclinic (Et_3_NH)_3_(Bi_1–*x*_Sb_*x*_)_2_Br_9_ solid solutions, we employed an identical synthetic procedure as in the synthesis of the triclinic Bi phase, replacing bismuth­(III) oxide with antimony­(III) oxide. Interestingly, in an attempt to synthesize a pure Sb compound (Et_3_NH)_3_Sb_2_Br_9_ we first obtained a side product (Et_3_NH)­SbBr_4_. After removing the crystals of (Et_3_NH)­SbBr_4_ from the mother liquor by filtration, phase pure triclinic (Et_3_NH)_3_Sb_2_Br_9_ crystals precipitate upon further evaporation. Our synthetic efforts showed that (Et_3_NH)_3_(Bi_1–*x*_Sb_*x*_)_2_Br_9_ solid solutions with triclinic phase structure form in the entire concentration range, 0 ≤ *x* ≤ 1.

(Et_3_NH)_3_Bi_2_Br_9_ crystallizes in two different structures with the noncentrosymmetric triclinic *P*1 and centrosymmetric monoclinic *P*2_1_/*c* space groups (Figure 1, Table S1). The triclinic phase has unit cell parameters *a* = 10.3311(4) Å, *b* = 12.2455(5) Å, *c* = 17.2778(7) Å, and angles α = 107.054(1)°, β = 94.566(1)°, and γ = 107.561(1)° (*R*_1_ = 3.71%), whereas the monoclinic phase crystallizes in a cell with parameters *a* = 23.216(3) Å, *b* = 20.304(2) Å, *c* = 17.6748(19) Å, and β = 109.648(4)° (*R*_1_ = 9.47%). Both phases are built of isolated [Bi_2_Br_9_]^3–^ dimeric structural units, which are composed of face-sharing distorted BiBr_6_ octahedra (Figure 1c). The distortion of the octahedral units results in a significant variation of the Bi–Br bond distances from 2.68 to 3.19 Å. The longest bond lengths correspond to the bridging Bi–Br bonds, indicating a considerable deviation from ideal octahedral geometry. The charge of the [Bi_2_Br_9_]^3–^ units is balanced by the organic cations Et_3_NH^+^, forming a 3D ionic structure in both polymorphs. The difference in the symmetry of the structures originates from the spatial arrangement of these structural units. Since the octahedra in the dimer structural unit have a shared face, these dimers are not centrosymmetric. Accordingly, the [Bi_2_Br_9_]^3–^ units are related by an inversion center in the *P*2_1_/*c* structure, whereas they are distributed in a less concerted fashion in the *P*1 structure (Figure 1). Since no species in (Et_3_NH)_3_Bi_2_Br_9_ drives the formation of enantiopure phases (e.g., there are no chiral organic cations), one would expect this compound to form a racemic mixture of crystals. However, all studied crystals of the triclinic structure consistently exhibited the same absolute configuration, as evidenced by the Flack parameter.^68^ This finding suggests potential factors such as kinetic control, crystallization conditions, or intermolecular interactions that may favor one enantiomer over the other. Detailed information on the structure refinement process for each dataset is provided in Table S2.

**Figure 1:**
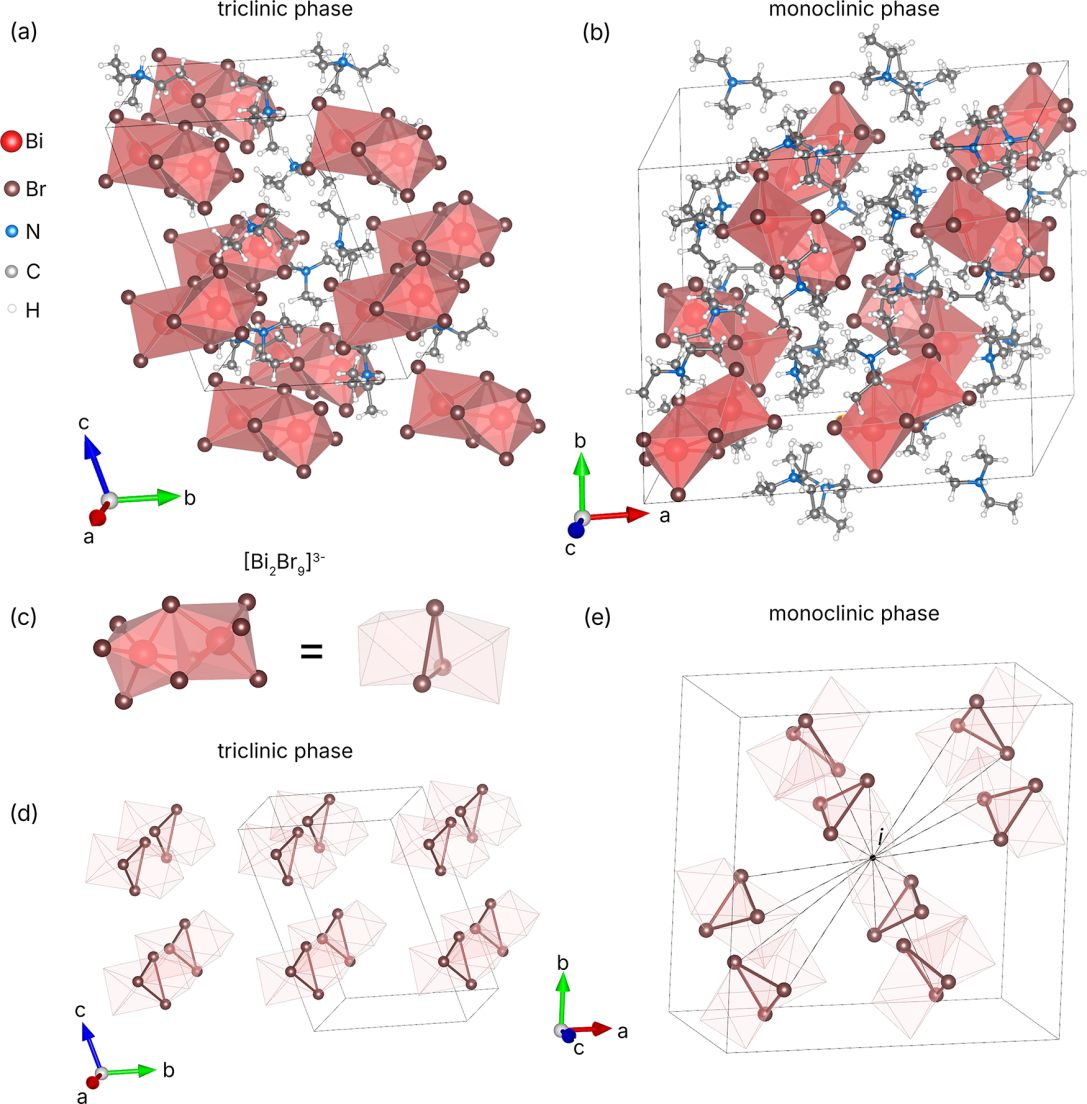
A view on the structure of (a) triclinic and (b) monoclinic polymorphs of (Et_3_NH)_3_Bi_2_Br_9_. (c) The main [Bi_2_Br_9_]^3–^ structural unit and its organization into (d) triclinic and (e) monoclinic phases.

Since the arrangement of the [Bi_2_Br_9_ ]^3–^ units defines the nonlinear optical properties of these phases, it is essential to consider structural unit alignment in both structures in greater detail. In the triclinic phase, all structural units are almost perfectly coaligned (Figure 1d) and are oriented uniformly throughout the crystal, which explains the noncentrosymmetry of the structure. The Sb-based triclinic phase has an identical structure, and a gradual decrease in parameters is also observed due to the difference in radii Sb^3+^ (0.76 Å) and Bi^3+^ (1.03 Å). In the monoclinic phase, the [Bi_2_Br_9_]^3–^ units are arranged in a centrosymmetric manner relative to each other (Figure 1e), leading to an overall centrosymmetric crystal structure. The four times larger unit cell observed in this phase suggests that achieving centrosymmetry imposes additional constraints on the spatial arrangement of the [Bi_2_Br_9_]^3–^ units. Specifically, each unit must be positioned and oriented such that it is related to another by an inversion center, which often requires a larger unit cell to accommodate the symmetry operations. In contrast, the noncentrosymmetric organization in the triclinic phase allows for a more compact and uniform orientation of the [Bi_2_Br_9_]^3–^ units, resulting in a smaller unit cell.

We studied the thermal behavior of the triclinic (Et_3_NH)_3_Bi_2_Br_9_ to probe its potential conversion to the monoclinic phase. Thermal analysis revealed three phase transitions at the onset temperatures of 90, 97, and 115 °C, along with a melting point at 135 °C (Figure 2a). When heated significantly above the melting point in an inert N_2_ atmosphere, the sample decomposed above 310 °C (Figure 2b). Phase transitions were confirmed using high-temperature powder X-ray diffraction (PXRD). According to the data of high-temperature PXRD, phase transitions occur at temperatures of 75, 85, and 110 °C (Figure 2c), which is consistent with the data of thermal analysis. The temperature shift of the phase transitions to the lower temperature region in the case of high-temperature diffraction is due to a significantly slower heating rate of the sample, which makes it possible to achieve equilibrium. Although we did not succeed in indexing the high-temperature PXRD patterns, the pattern calculated from the monoclinic phase did not match with any of the high-temperature experimental patterns, indicating that these correspond to distinct high-temperature polymorphic modifications.

**Figure 2:**
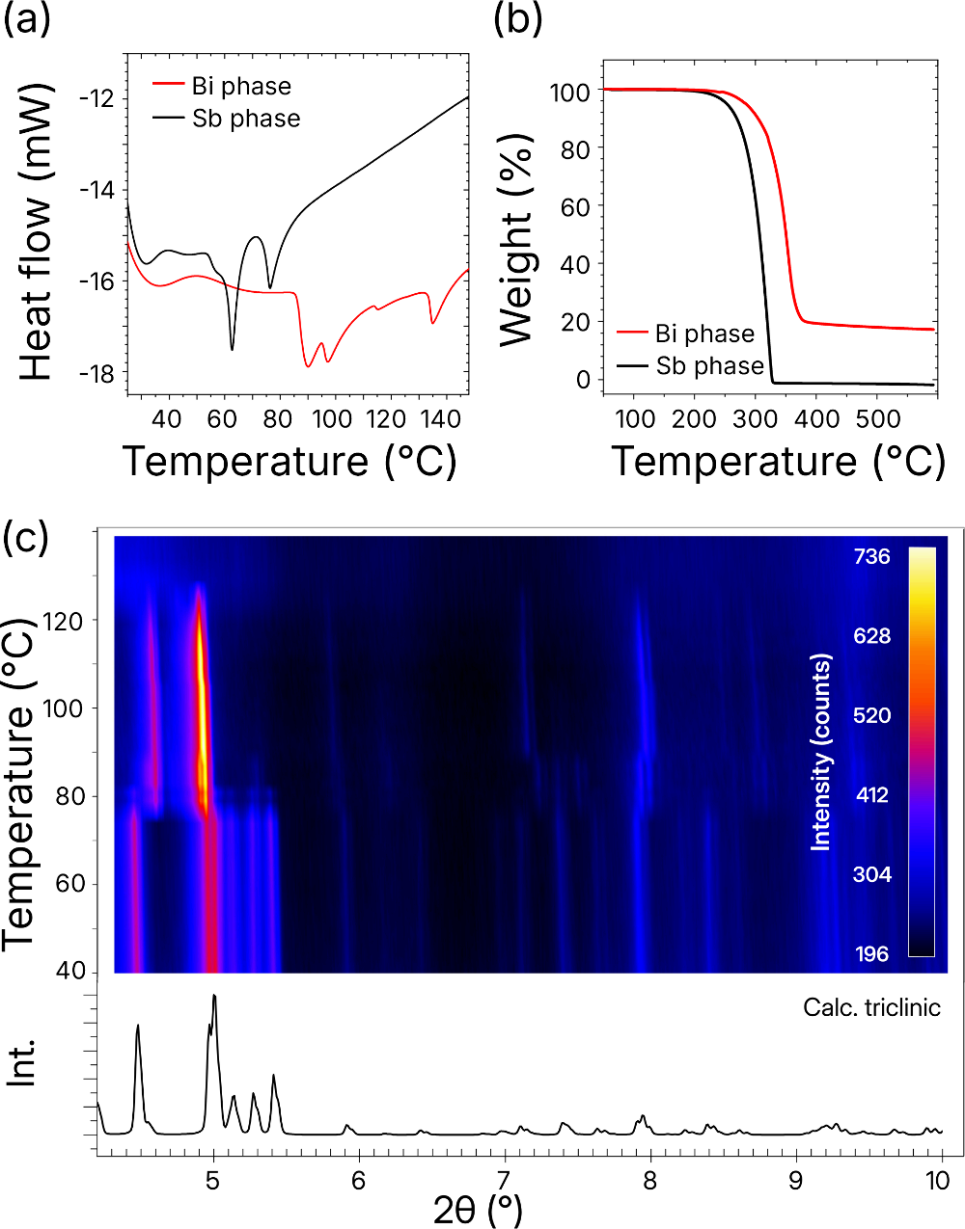
Thermal behavior of (Et_3_NH)_3_Bi_2_Br_9_ and (Et_3_NH)_3_Sb_2_Br_9_. (a) Differential scanning calorimetry (DSC) and (b) thermal gravimetric analysis (TGA) of both samples. (c) Waterfall plot of temperature-dependent PXRD results for the triclinic phase showing two structural phase transitions and melting of the sample.

Thermal analysis of Sb-containing phases showed a nonlinear decrease in the temperature of all phase transitions, including the melting point (Figures 2a and 2b). The low melting point of the pure antimony compound (Et_3_NH)_3_Sb_2_Br_9_ of 75 °C suggests that this compound is an ionic liquid.^63^ The onset of the decomposition process also shifts to lower temperatures with an increase in the amount of antimony, which is 282.5 °C for the pure antimony compound. Interestingly, the Sb compound decomposes leaving no residue behind, i.e., with zero residual weight, indicating that likely decomposition products are volatile Et_3_N, SbBr_3_, and HBr. Based on the thermal analysis of (Et_3_NH)_3_Bi_2–*x*_Sb_*x*_Br_9_, one can conclude that this solid solution series can form ionic liquids with compositionally tunable melting points and a relatively wide operational range of ∼200 °C.

Another interesting fact is the possibility of converting these Bi^3+^ phases into one another by controlling the cooling rate of the melt in the air. Notably, the molten state is typically out of reach for synthesizing hybrid materials due to their low thermal stability. However, in the case of these two polymorphs, this transformation is possible because of the relatively low melting points (130 °C for (Et_3_NH)_3_Bi_2_Br_9_). We observed that slow cooling of a molten mixture of the two polymorphs from 150 °C to room temperature at a rate of 5–10 °C/h results in the formation of a phase pure sample of the triclinic polymorph (Figure 3). The cooling rate is crucial, and it depends on the mass of the initial sample: bulkier samples require slower cooling rates to achieve a pure triclinic phase. On the other hand, fast cooling rates or temperature quenching lead to the formation of a mixture of the triclinic and monoclinic phases, even when a phase-pure triclinic sample was used as a starting material (Figure 3). We could not obtain a phase-pure monoclinic sample from the melt, regardless of how quickly the sample was cooled, indicating its metastable nature at room temperature. Our DFT calculations showed that both phases have very close energies of −4.9924 and −4.9929 eV/atom for the triclinic and monoclinic phases, respectively, confirming the experimental observation that both polymorphs can form under similar conditions. This distinction between the two phases highlights the role of kinetic versus thermodynamic control in their formation, where the formation of the triclinic phase is favored under equilibrium conditions, whereas the monoclinic one results from nonequilibrium, fast-crystallization processes. The possibility of converting the phase mixture into the pure triclinic phase opens up the rare, for hybrid materials, possibility of melt processing for different applications, including SHG.

**Figure 3:**
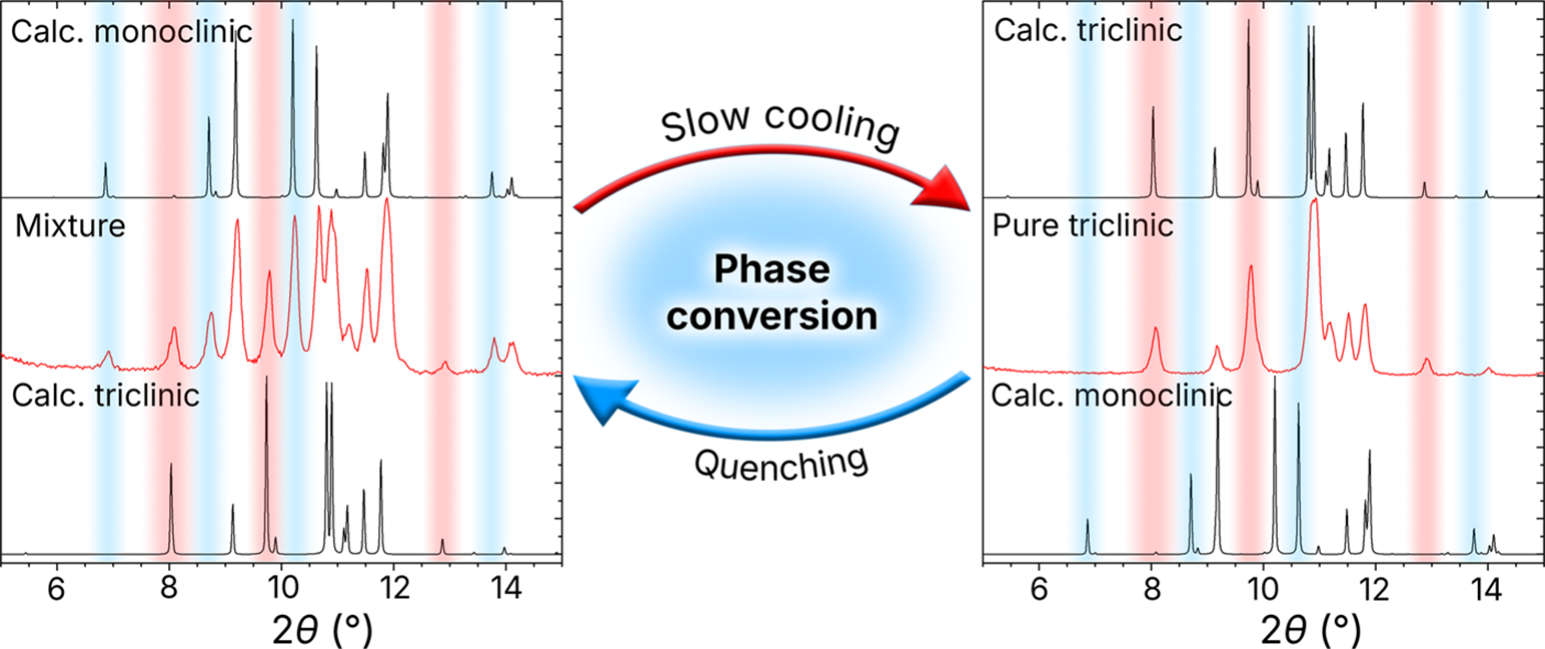
Temperature-assisted conversion between the polymorphs of (Et_3_NH)_3_Bi_2_Br_9_. Slow cooling of a (Et_3_NH)_3_Bi_2_Br_9_ melt promotes the crystallization of a phase pure triclinic phase, whereas fast cooling or quenching enables the formation of a metastable monoclinic phase as a side product.

We characterized the optical properties of the triclinic (Et_3_NH)_3_Bi_2_Br_9_ by measuring UV–vis, Raman, and IR spectra. The optical bandgap of 2.6 eV was determined from UV–vis reflectance data (Figure S2) and agrees well with the yellow color of the crystals. An onset of absorption above 2.25 eV can indicate the presence of defect states or an indirect nature of the bandgap. FTIR spectrum analysis of the triclinic phase shows characteristic N–H and C–H stretching vibrations in a 3000–2800 cm^–1^ region, C–N stretching bands in 1000–1200 cm^–1^ region, and bending, rocking, and wagging modes from the C–H groups, C–C and C–N bonds below 1000 cm^–1^, as it is shown in Figure S3. Interestingly, N–H stretching band is also present on the spectrum in the range of 3000–3200 cm^–1^, which occurs in tertiary amines if they are protonated.

The nonlinear optical properties of the triclinic phase were evaluated using second-harmonic generation measurements. The SHG responses were examined using both infrared (2090 nm) and near-infrared (1064 nm) laser excitation sources, with AgGaS_2_ (AGS) and KH_2_PO_4_ (KDP) employed as reference materials, respectively. As shown in Figure 4b, the triclinic phase exhibits a moderate SHG intensity at 2090 nm, reaching approximately 0.08 × AGS. Although lower in intensity compared to previously reported hybrid materials (0.53–1.3 × AGS),^64–66^ the observed phase matchability at this wavelength underscores its potential applicability in IR nonlinear optical devices (Figure 4a). Additionally, at 1064 nm excitation, the triclinic phase demonstrates a higher SHG intensity, approximately 1.29 × KDP with particle size 63 μm, although without phase matching. These results highlight the importance of spectral tuning for hybrid SHG materials and suggest this triclinic polymorph as a promising candidate for specific IR-region nonlinear optical applications.

**Figure 4:**
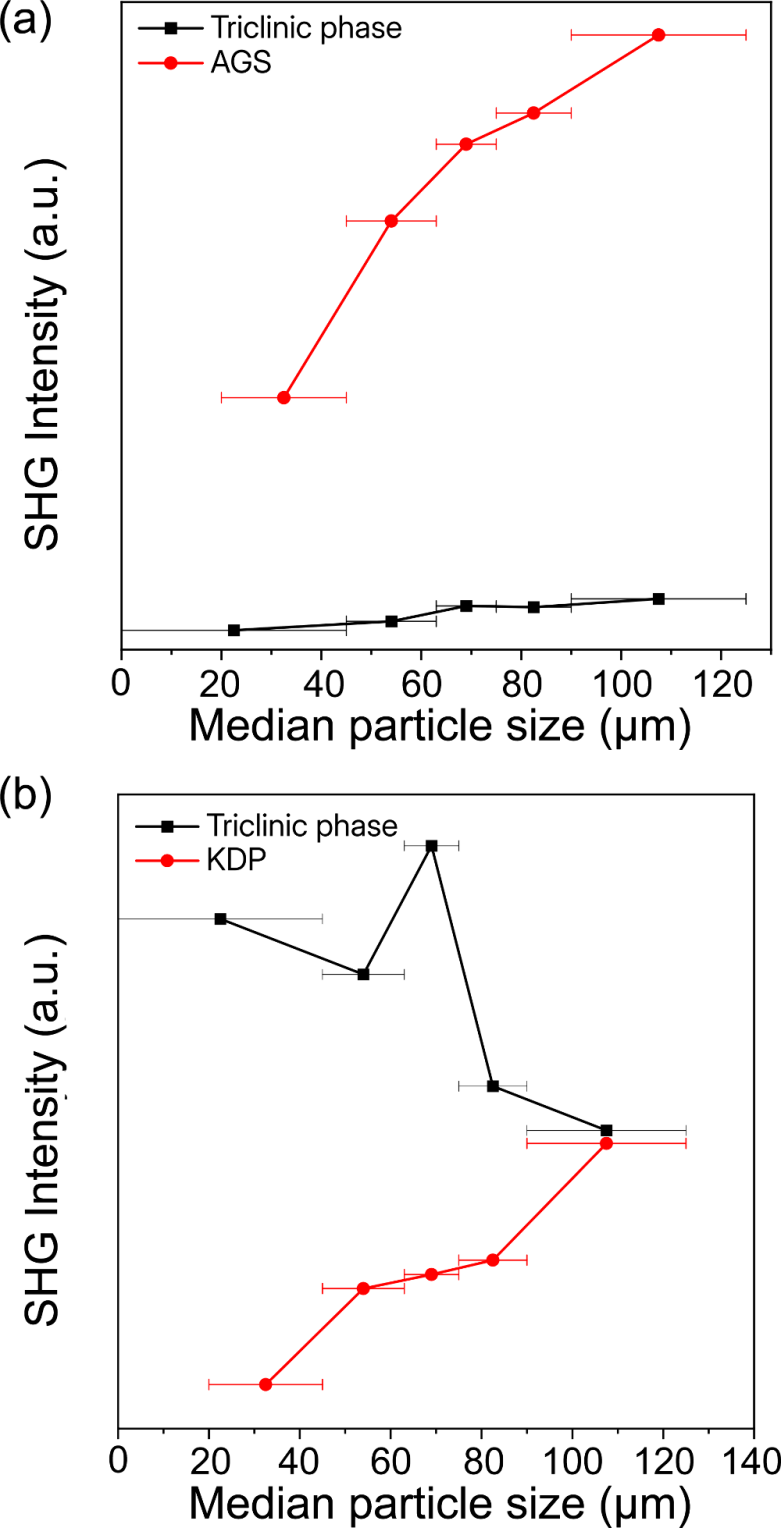
SHG response of the triclinic phase compared to (a) AgGaS_2_ at a laser wavelength of 2090 nm and (b) KH_2_PO_4_ at a laser wavelength of 1064 nm.

In conclusion, the formation of structurally distinct polymorphs of (Et_3_NH)_3_Bi_2_Br_9_ was achieved through the solution method with controlled evaporation. The arrangement of noncentrosymmetric [Bi_2_Br_9_]^3–^ units leads to the formation of two polymorphs, with the triclinic phase exhibiting an SHG response both in the IR and visible regions. The temperature-dependent phase transitions and low melting points of these phases offer unique opportunities for hybrid materials for melt-processing, further expanding their potential applications.

## Supplementary Material









## Data Availability

CCDC Depository Nos. 2411445, 2411449, and 2411450 contain the supplementary crystallographic data for this paper. These data can be obtained free of charge via www.ccdc.cam.ac.uk/data_request/cif, or by emailing data_request@ccdc.cam.ac.uk, or by contacting The Cambridge Crystallographic Data Centre, 12 Union Road, Cambridge CB2 1EZ, UK; fax: + 44 1223 336033.

## Notes

Experimental data, crystallographic data and XRD experiment parameters, atomic displacement parameters, bond lengths, bond angles, Tauc plot, and Raman spectra (PDF)
(CIF)
(CIF)
(CIF)
