# Supplementary material for: Hybrid Bismuth Halide with Rich Polymorphism and Second Harmonic Generation Response
Source: ACS Mater Lett. 2025 Jul 7;7(8):2814–21. doi: 10.1021/acsmaterialslett.5c00784 (PMC12326387; doi:10.1021/acsmaterialslett.5c00784)
Supplement: Supplementary file 1 [file tz5c00784_si_001.pdf]

Supporting information for

Hybrid Bismuth Halide with Rich Polymorphism and Second Harmonic Generation Response

Aleksandra D. Valueva,[a] Sergei A. Novikov,[a] Eric Gabilondo,[b] Hunter B. Tisdale,[c] Alevtina A. Maksimova,[c] Mikhail Parker,[a] Vladimir Reukov,[d] and Vladislav V. Klepov*[a]

[a]Department of Chemistry, University of Georgia, Athens, Georgia, 30602, USA

[b]Department of Chemistry, University of Houston, Houston, Texas, 77204, USA

[c]Department of Chemistry, University of South Carolina, Columbia, South Carolina, 29208, USA

[d]Textiles, Merchandising and Interiors, University of Georgia, Athens, Georgia, 30605, USA

Table of Contents

Experimental details S3-S4
Figure S1. PXRD patterns of synthesized samples. S5
Figure S2. Tauc Plot of triclinic phase. S6
Figure S3. FTIR spectra of triclinic phase. S7
Table S1. Crystal data and structure refinement of triclinic and monoclinic phases. S8
Table S2. Flack parameters. S9

Experimental details

Reagents. Commercially available Bi_2O_3 (Thermo Scientific, 99.9995 %), Sb_2O_3 (Thermo Scientific, 99.6 %), Triethylamine (BeanTown Chemical, 99 %), HBr (48 wt. % water solution, Thermo Scientific, pure) were used as received.

Synthesis of $(Et_3NH)_3Bi_2Br_9$ as a mixture of *P*1 and *P*2$_1$/*c* polymorphs. Bi_2O_3 (250 mg, 0.537 mmol) and Et_3N (225 μL, 1.61 mmol) were dissolved in HBr (1.5 mL, 48 wt.%) in a 20 mL scintillation vial. The mixture was continuously stirred until it became clear and placed in the oven at 95 ^oC for 1 hour to ensure complete dissolution of Bi_2O_3. The solution was left open to evaporate at room temperature, or left in the oven. Pale yellow crystals formed and were collected by vacuum filtration. According to X-ray diffraction data, the resulting product consisted of a mixture of the *P*1 and *P*2$_1$/*c* polymorphs (fast evaporation), or of the *P1* polymorph exclusively (slow evaporation).

Conversion of the *P*1 and *P*2$_1$/*c* polymorphs mixture to pure *P*1 polymorph. The sample with a mixture of *P*1 and *P*2$_1$/c polymorphs was dried under vacuum for 3 days. The dry sample was placed in a silica tube, and sealed under vacuum. The tube with a sample was placed in an oven, rapidly heated up to 150°C to ensure a complete sample melting, and slowly cooled down at a rate of 5-10°C/h.

Powder X-ray diffraction (PXRD). PXRD data were collected on a Bruker D2 PHASER diffractometer using Cu Kα radiation (λ = 1.5418 Å) generated at a power of 30 kV and 10 mA over a 2θ range 10−45° with a step size of 0.01° and data acquisition time of 2.000 s/step.

High temperature powder X-ray diffraction (HT-PXRD). HT-PXRD on a sample of the *P*1 polymorph was conducted on a Rigaku SmartLab 9kW equipped with a rotating Mo anode utilizing cross-beam (Debye-Scherrer geometry) optics, a D/teX Ultra 250 HE detector, and an Anton Paar HTK1200N high-temperature oven chamber with a capillary attachment. Mo Kβ X-rays were filtered out using a Zr filter and the remaining Mo $K\alpha_1$ and $K\alpha_2$ X-rays were utilized for measurements. Data were collected on a sample loaded into a borosilicate capillary that was loaded into the oven chamber and rotated in situ. PXRD patterns were taken from 4 to 10° 2θ at 0.2 °/min every 5 °C starting at 40 °C and up to 80 °C, then every 2 °C up to 120 °C, and finally every 5 °C up to 140 °C.

Single crystal X-ray diffraction. Single crystal X-ray diffraction experiments (Mo Kα radiation) were performed on a Bruker D8 QUEST diffractometer equipped with a PHOTON 100 CMOS area detector at room temperature. Data integration was performed via SAINT-Plus software[1]; absorption correction was done with the SADABS program[2]. Structures of new bismuth hybrid bromides were solved by the intrinsic phasing method (SHELXT[3], Olex2[4]) and refined by the full-matrix least squares method against F^2 in an anisotropic approximation (SHELXL[5]). Hydrogen atoms were placed in geometrically calculated positions.

Differential Scanning Calorimetry (DSC) and Thermogravimetric Analysis (TGA). DSC/TGA measurements were performed on a polycrystalline powder samples using a SDT Q600 Thermogravimetric Analyzer and an alumina pan as the sample holder. The samples were heated from room temperature to the target temperature 600 °C at 10 °C/min under a flow of nitrogen gas (100 mL/min).

DFT calculations. First-principles calculations were performed using density functional theory (DFT) with the Vienna Ab-initio Package (VASP) planewave code,[6,7] generalized gradient approximation of Perdew, Burke and Ernzerhof (PBE),[8] and projector augmented wave (PAW) method.[9,10] The initial unit cells were converted to a primitive cell using VESTA software before geometry optimization.[11] The ground state geometries at 0 K were optimized by relaxing the cell volume, atomic positions, and cell symmetry until the maximum force on each atom is less than 0.01 eV/Å. Non-spin-polarized calculations were performed, with 520 eV cut-off energy for the plane wave basis set and,, 10^{-5} eV energy convergence criteria.

SHG measurements. Room-temperature mid-infrared (IR) SHG data were measured using a modified Kurtz–Perry system with a Ho:YAG laser at the wavelength of 2.09 μm.[12] The finely ground polycrystalline powders were sieved to 63–75 μm and measured with AgGaS2 (AGS) as the reference sample. A photomultiplier tube was used to collect the intensity of the frequency-doubled output data emitted from the sieved powdered samples.

Optical properties measurements. Optical reflectance measurements were performed at room temperature using a Shimadzu UV-2450 (Kyoto, Japan) spectrometer operating in a wavelength range from 200 to 800 nm. $BaSO_4$ was used as a non-absorbing reflectance reference.

Biological activity. Since some hybrid materials demonstrate promising antibacterial activity, we probed $(Et_3NH)_3Bi_2Br_9$ properties using bacterial inhibition assay against E. coli and S. aureus.[13] Without the triclinic phase presence in the bacterial suspension, the bacterial growth was unrestricted, with the number of colony-forming units (CFU) for both bacterial strains exceeding 6000. However, at 0.1 mg/100 μL concentration of the triclinic phase, the numbers of CFUs were significantly reduced, with mean counts of 159 for E. coli and 699 for S. aureus. At 1 mg/100 μL concentration, the triclinic phase exhibited dose-dependent enhancement in activity, with CFU counts reduced to 87 and 226 for E. coli and S. aureus, respectively.

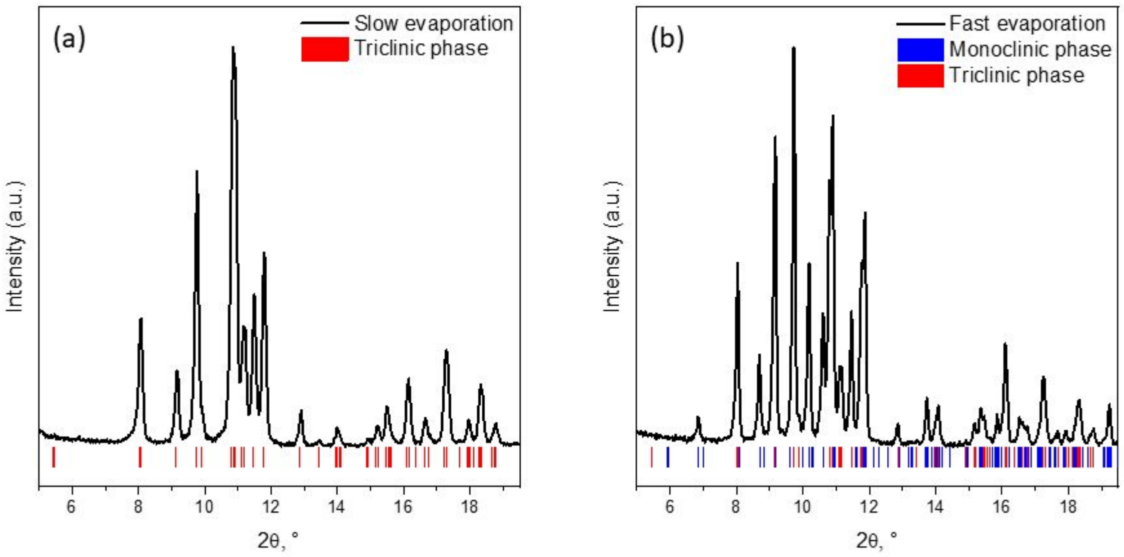

Figure S1. (a) PXRD of the triclinic phase after synthesis. (b) PXRD pattern of a mixture of the triclinic and monoclinic phases after synthesis.

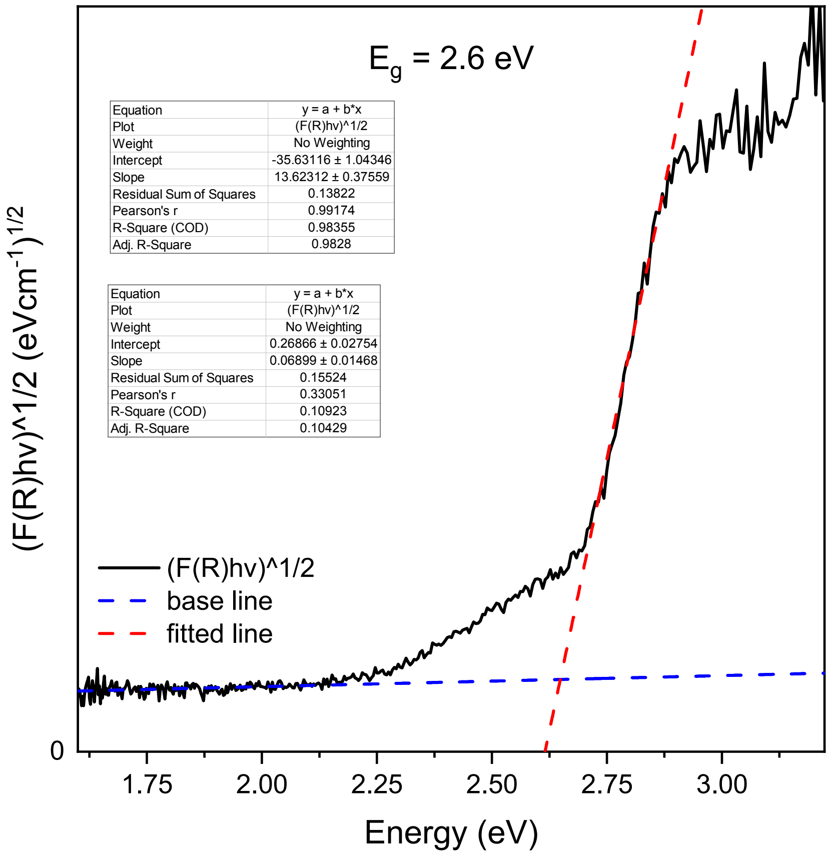

Figure S2. Band gap calculation for the triclinic phase.

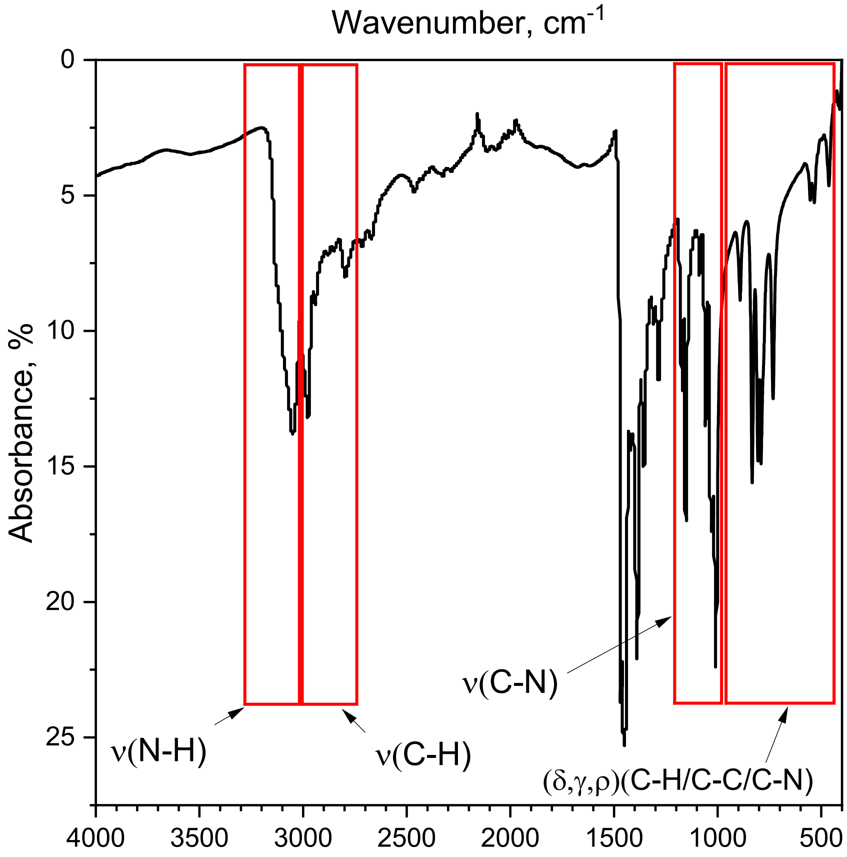

Figure S3. FTIR spectra of the triclinic phase.

Table S1. Crystal data and structure refinement for triclinic $(Et_3NH)_3(Bi/Sb)_2Br_9$ and monoclinic $(Et_3NH)_3Bi_2Br_9$ polymorphs.

Empirical formula	$C_{18}H_{48}Bi_2Br_9N_3$	$C_{18}H_{48}Bi_2Br_9N_3$	$C_{18}H_{48}Sb_2Br_9N_3$
Formula weight	1443.74	2887.48	1269.28
Temperature/K	299	299	301
Crystal system	triclinic	monoclinic	triclinic
Space group	P1	$P2_1/c$	P1
a/Å	10.3311(4)	23.216(3)	10.2812(6)
b/Å	12.2455(5)	20.304(2)	12.1821(7)
c/Å	17.2778(7)	17.6748(19)	17.2354(10)
α/°	107.0540(10)	90	107.073(2)
β/°	94.5660(10)	109.648(4)	94.018(2)
γ/°	107.5610(10)	90	107.737(2)
Volume/$Å^3$	1958.13(14)	7846.6(15)	1935.1(2)
Z	2	8	2
$\rho_{calc}g/cm^3$	2.449	2.444	2.178
μ/mm^{-1}	18.17	18.137	10.701
F(000)	1316	5264	1188
Crystal size/mm^3	0.15 × 0.09 × 0.07	0.2 × 0.16 × 0.08	0.09 × 0.06 × 0.04
Radiation	MoKα (λ = 0.71073)	MoKα (λ = 0.71073)	MoKα (λ = 0.71073)
2Θ range for data collection/°	3.714 to 61.264	4.424 to 50	4.58 to 60
Index ranges	-14 ≤ h ≤ 14, -17 ≤ k ≤ 17, -23 ≤ l ≤ 24	-27 ≤ h ≤ 27, -24 ≤ k ≤ 24, -21 ≤ l ≤ 21	-14 ≤ h ≤ 14, -17 ≤ k ≤ 17, -24 ≤ l ≤ 24
Reflections collected	141739	172030	126343
Independent reflections	23178 [R_{int} = 0.0407, R_{sigma} = 0.0433]	13813 [R_{int} = 0.1555, R_{sigma} = 0.0681]	21932 [R_{int} = 0.0719, R_{sigma} = 0.0740]
Data/restraints/parameters	23178/14/581	13813/0/595	21932/7/597
Goodness-of-fit on F^2	1.023	1.042	1.001
Final R indexes [I>=2σ (I)]	R_1 = 0.0371, wR_2 = 0.0857	R_1 = 0.0512, wR_2 = 0.0905	R_1 = 0.0402, wR_2 = 0.0644
Final R indexes [all data]	R_1 = 0.0603, wR_2 = 0.0937	R_1 = 0.0995, wR_2 = 0.1095	R_1 = 0.1099, wR_2 = 0.0821
Largest diff. peak/hole / e $Å^{-3}$	1.46/-1.21	1.99/-2.47	0.85/-0.75
Flack parameter	-0.010(6)		0.012(8)

Table S2. Flack parameters of different crystals of triclinic phase

Name of the sample	No of crystal	Flack parameter
AV_33a	1	-0.054(17)
AV_33a	2	-0.051(8)
AV_33a	3	-0.039(13)
AV_33a	4	0.14(2)
AV_34h	1	-0.047(11)
AV_34h	2	-0.07(4)
AV_34h	3	-0.010(6)

References

(1) SAINT-Plus (Version 7.68). *Bruker AXS Inc., Madison, Wisconsin, USA.* **2007**.

(2) SADABS. *Bruker AXS Inc., Madison, Wisconsin, USA.* **2008**.

(3) Sheldrick, G. M. *SHELXT* – Integrated Space-Group and Crystal-Structure Determination. *Acta Crystallographica Section A Foundations and Advances* **2015**, *71*, 3–8. https://doi.org/10.1107/S2053273314026370.

(4) Dolomanov, O. V.; Bourhis, L. J.; Gildea, R. J.; Howard, J. A. K.; Puschmann, H. *OLEX2* : A Complete Structure Solution, Refinement and Analysis Program. *Journal of Applied Crystallography* **2009**, *42*, 339–341. https://doi.org/10.1107/S0021889808042726.

(5) Sheldrick, G. M. Crystal Structure Refinement with *SHELXL*. *Acta Crystallographica Section C Structural Chemistry* **2015**, *71*, 3–8. https://doi.org/10.1107/S2053229614024218.

(6) Kresse, G.; Furthmüller, J. Efficient Iterative Schemes for *Ab Initio* Total-Energy Calculations Using a Plane-Wave Basis Set. *Physical Review B* **1996**, *54*, 11169–11186. https://doi.org/10.1103/PhysRevB.54.11169.

(7) Kresse, G.; Furthmüller, J. Efficiency of Ab-Initio Total Energy Calculations for Metals and Semiconductors Using a Plane-Wave Basis Set. *Computational Materials Science* **1996**, *6*, 15–50. https://doi.org/10.1016/0927-0256(96)00008-0.

(8) Perdew, J. P.; Burke, K.; Ernzerhof, M. Generalized Gradient Approximation Made Simple. *Physical Review Letters* **1996**, *77*, 3865–3868. https://doi.org/10.1103/PhysRevLett.77.3865.

(9) Blöchl, P. E. Projector Augmented-Wave Method. *Physical Review B* **1994**, *50*, 17953–17979. https://doi.org/10.1103/PhysRevB.50.17953.

(10) Kresse, G.; Joubert, D. From Ultrasoft Pseudopotentials to the Projector Augmented-Wave Method. *Physical Review B* **1999**, *59*, 1758–1775. https://doi.org/10.1103/PhysRevB.59.1758.

(11) Momma, K.; Izumi, F. *VESTA 3* for Three-Dimensional Visualization of Crystal, Volumetric and Morphology Data. *Journal of Applied Crystallography* **2011**, *44*, 1272–1276. https://doi.org/10.1107/S0021889811038970.

(12) Kurtz, S. K.; Perry, T. T. A Powder Technique for the Evaluation of Nonlinear Optical Materials. *Journal of Applied Physics* **1968**, *39*, 3798–3813. https://doi.org/10.1063/1.1656857.

(13) Azmy, A.; Zhao, X.; Angeli, G. K.; Welton, C.; Raval, P.; Wojtas, L.; Zibouche, N.; Manjunatha Reddy, G. N.; Trikalitis, P. N.; Cai, J.; Spanopoulos, I. One-Year Water-Stable and Porous Bi(III) Halide Semiconductor with Broad-Spectrum Antibacterial Performance. *ACS Applied Materials & Interfaces* **2023**, *15*, 42717–42729. https://doi.org/10.1021/acsami.3c06394.
